# Dissecting the molecular interactions between botanical extracts and the human gut microbiota

**DOI:** 10.3389/fmicb.2025.1610170

**Published:** 2025-07-16

**Authors:** Leonardo Mancabelli, Chiara Tarracchini, Giulia Longhi, Giulia Alessandri, Marco Ventura, Francesca Turroni

**Affiliations:** ^1^Department of Medicine and Surgery, University of Parma, Parma, Italy; ^2^Microbiome Research Hub, University of Parma, Parma, Italy; ^3^Laboratory of Probiogenomics, Department of Chemistry, Life Sciences, and Environmental Sustainability, University of Parma, Parma, Italy

**Keywords:** gut microbiota, microbiome, dandelion root, aloe vera gel, bifidobacteria

## Abstract

Over millions of years, humans and their gut microbes have developed a symbiotic relationship that benefits both organisms. Many plants and herbs consumed as food by humans, such as aloe vera gel and dandelion root extracts, contain bioactive compounds with recognized therapeutic or preventive effects. However, the impact of these botanicals on the composition and functionality of the human gut microbiota is not yet understood. In this study, the molecular impact of these botanicals on reconstructed human gut microbiota was assessed by *in*-*vitro* bioreactor experiments followed by metagenomics and transcriptomic approaches, highlighting both taxonomic and functional changes in the human gut microbiome. Furthermore, cross-feeding activities established by common human gut microbial taxa like *Bacteroides* spp. when cultivated on these extracts were assessed. In conclusion, the results show that botanicals affect intestinal populations that are highly dependent on the microbial taxa present and that trophic interactions are established in few key gut members.

## 1 Introduction

The human intestine hosts one of the most complex, diverse, and intricate microbial communities in the biosphere, known as the gut microbiota, which includes bacteria, Archaea, viruses, fungi, and protozoa (Ursell et al., 2012; Matijasic et al., 2020; Sudheer et al., 2022). Millions of years of co-evolution between the human host and its gut microbial ecosystem have led to the establishment of a binary symbiotic relationship from which both organisms benefit (Pickard et al., 2017; Takiishi et al., 2017; Alessandri et al., 2019). Indeed, the host guarantees a wide variety of nutrients and a perfect environment for the proliferation of gut microbes, while the gut microbiota, in turn, offers the host a plethora of metabolic and physiological activities that play a crucial role in influencing host health (Pickard et al., 2017; Takiishi et al., 2017; Afzaal et al., 2022; Lee et al., 2024). In addition to its ability to metabolize indigestible food compounds, it can provide nutrition for enterocytes and produce a wide variety of bioactive metabolites, including short-chain fatty acids (SCFAs), vitamins, neuroactive molecules, tryptophan derivatives, and indole lactic acids (Rahman et al., 2023; Tarracchini et al., 2024). The intestinal microbiota is also involved in a continuous molecular dialogue with the host immune system, influencing immune responses as well as bowel homeostasis and functionality (Levy et al., 2017; Rahman et al., 2023; Donkers et al., 2024; Tarracchini et al., 2024). Furthermore, it is widely recognized that the gut microbiota is important in limiting pathogen colonization and promoting the maintenance of intestinal barrier integrity (Moens and Veldhoen, 2012; Pickard et al., 2017; Sanchez et al., 2017; Van Hul et al., 2024). However, despite these multiple beneficial activities exploited by gut microbes, the intestinal microbiota can also be responsible for adverse health effects. Indeed, when a disruption of the climax and dynamic balance, also known as homeostasis, among the bacterial species of the gut microbiota occurs, a dysbiosis condition can be established with consequent stimulation and exacerbation of the host's health disturbances (DeGruttola et al., 2016). This imbalance contributes to the onset of several chronic pathological conditions, including intestine-related pathologies such as inflammatory bowel disease, irritable bowel syndrome, or colorectal cancer, but also extra-intestinal diseases, ranging from diabetes and obesity to cardiovascular, kidney, liver, and even neurological disorders (Belizario and Faintuch, 2018; Wilkins et al., 2019; Dixit et al., 2021; Lee et al., 2024; Manske, 2024).

In this context, in recent decades, several efforts have been placed to identify strategies that can be adopted to maintain intestinal homeostasis or prevent/restore intestinal dysbiosis. Among many different approaches, functional and/or therapeutic foods are valid ways to prevent gut dysbiosis (Medina-Vera et al., 2019; Perez-Burillo et al., 2020; Banerjee et al., 2023). These foods may contain bioactive compounds such as tannins, polyphenols, flavonoids, terpenoids, and complex polysaccharides, which can have functional effects on our health (Thumann et al., 2019; Sudheer et al., 2022; Banerjee et al., 2023; Jacquier et al., 2024). Within botanicals with possible effects on the human gut microbiota, aloe vera gel and dandelion root extracts have been used for centuries as herbal medicines due to their therapeutic benefits (Le Phan et al., 2021; Cuvas-Limon et al., 2022; Li et al., 2022; Maiuolo et al., 2022; Li et al., 2024; Yan and Dong, 2024). Specifically, dandelion root (DR) extracts have renowned beneficial effects on the gastrointestinal tract, reducing the expression of reactive proteins and oxidative stress and regulating gut microbiota through their antioxidant and anti-inflammatory properties (Kaur et al., 2021; Li et al., 2022; Yan and Dong, 2024). In parallel, aloe vera gel (AVG), which is currently included in several beverages with functional potential, is used for its countless beneficial effects on human health, encompassing immunomodulatory, anti-obesity, antiviral, anti-diabetic, and antibacterial properties (Holscher et al., 2015; Cuvas-Limon et al., 2022; Maiuolo et al., 2022). At the same time, certain studies demonstrated the role of these medicinal plant extracts in regulating gut dysbiosis (Fu et al., 2022; Maiuolo et al., 2022; Yan et al., 2023; Yan and Dong, 2024). However, despite the multiple beneficial properties of AVG and dandelion root extracts, these botanicals' effect on the taxonomic composition and functional potential of the human gut microbiota is still far from being thoroughly dissected.

Here, an *in-vitro* growth experiment involving 10 artificial gut microbiota was performed to investigate whether AVG and dandelion root extracts could have a role in modulating both the taxonomic and functional profile of the human intestinal ecosystem through the combination of metagenomic approaches with flow cytometry. In addition, specific cross-feeding activities of the two botanicals on the most impacted gut microbes were evaluated.

## 2 Materials and methods

### 2.1 *In-vitro* gut microbiota cultivation in the presence of aloe vera gel or dandelion root extracts

To evaluate whether AVG or dandelion root can modulate the taxonomic composition of the human gut microbiota, 10 artificial gut microbiota (AGM) communities were cultivated in the presence of two botanical extracts. Specifically, the AGMs were obtained in the framework of another study (Alessandri et al., 2024). Briefly, the bacterial community of each collected fecal sample was immobilized on 1–2 mm gellan-xanthan gel beads and inoculated in a bioreactor-based colonic fermentation system (Solaris Biotech Solutions, Italy). AGM cultivation was performed by using a human gut environment-simulating growth medium, which has been previously described (Alessandri et al., 2022), with a temperature set at 37°C, continuous stirring at 200 rpm, while the pH was maintained at 6.8 by the addition of 2.5 M NaOH. The cultivation was run for 15 days in a continuous mode (Alessandri et al., 2024). After the gut microbiota stabilization, an aliquot of the obtained AGMs was withdrawn from the bioreactor system and used as inoculum for subsequent experiments. Specifically, each AGM was individually cultivated in the presence of aloe vera gel or dandelion root extracts or without the addition of any botanicals as a control sample. AGMs were inoculated at a final inoculum concentration of 2% (v/v) in 2 mL of the same culture medium used to stabilize fecal samples (Alessandri et al., 2022), while botanical dry extracts, after filter-based sterilization, were added to the culture medium to a final concentration of 0.1% (w/v). Cultivations were performed following the MiPro model protocol (Li et al., 2019), i.e., 96-deep well plates were covered with a silicone gel mat with a vent hole on each well created with a sterile syringe needle to facilitate gas exchange. Plates were incubated under anaerobic conditions at 37°C and shaken at 5,000 rpm. After 24 h, cultures were collected and stored at −80°C until they were processed for DNA extraction and flow cytometry-based total bacterial cell count. Each experiment was carried out in triplicates.

### 2.2 DNA extraction and shallow shotgun sequencing

After growth assays in the presence of AVG or dandelion root extracts, every obtained replicate, including the control samples, was subjected to total bacterial DNA extraction using the QIAmp DNA Stool Mini Kit (Qiagen, Germany), following the manufacturer's instructions. Then, the extracted DNA was prepared using the Illumina Nextera XT DNA Library Preparation Kit, following the Illumina Nextera XT protocol. Specifically, DNA samples were enzymatically fragmented, barcoded, and purified using magnetic beads. Subsequently, the prepared samples were quantified using a fluorometric Qubit quantification system (Life Technologies, USA), loaded on a TapeStation Instrument (Agilent Technologies, USA), and normalized to 4 nM. Paired-end sequencing was performed using an Illumina NextSeq sequencer (Illumina Inc., San Diego, USA) with the NextSeq reagent Kit v3 and a 1% PhiX control library spike-in.

### 2.3 Shallow shotgun metagenomic dataset analysis

The obtained raw data in.fastq format were submitted to a filtering step to remove reads with a quality of < 25 as well as sequencing corresponding to human DNA by mapping the reads on the *Homo sapiens* genome, while reads with a length of >149 bp were retained. As previously reported, quality-filtered data was used for further analysis with METAnnotatorX2 for taxonomic profile reconstruction (Milani et al., 2021). Retained sequences were used as input to perform a MegaBLAST local alignment of reads to a pre-processed database, including available genomes of eukaryotes (Fungi and Protists), viruses, archaea, and bacteria, following the METAnnotatorX2 protocol (Milani et al., 2021). Reads showing a nucleotide identity of >94% to the genomes included in the database were classified at the species level, while they were classified at the genus level as undefined species when a lower percentage was detected. Taxonomic profiles of each sample were exploited to calculate the Shannon index and species richness to assess α-diversity. Species richness represented the number of bacterial species detected for each metagenomic sample. Bray-Curtis dissimilarity matrices based on species abundance, calculated through the Rstudios software, were used to evaluate similarities between samples (β-diversity). The similarity range was calculated as a value between 0 and 1. Principal coordinate analysis (PCoA) was used to represent β-diversity through Emperor. In the PCoA, each sphere represents a single sample distributed in tridimensional space according to its specific taxonomic profile. Functional profiling of the sequenced reads was performed with the METAnnotatorX2 bioinformatics platform (Milani et al., 2021). Functional classification of reads was carried out to identify metabolic pathways based on the MetaCyc database (release 24.1; Caspi et al., 2016) through RAPSearch2 software (Ye et al., 2011; Zhao et al., 2012).

### 2.4 Evaluation of bacterial cell density through flow cytometry

Each replicate underwent a total bacterial cell count through flow cytometry. Specifically, replicates were 10,000 diluted in Phosphate Buffered Saline (PBS) solution, and 1 mL of the bacterial dilution was stained with 1 μL of SYBR Green I (Thermo Fisher Scientific, USA; 1:100 diluted in DMSO; Merk, Germany), vortex-mixed, and incubated in the dark for at least 15 min before measurement. Count assays were performed using the Attune NxT flow cytometer (Thermo Fisher Scientific, USA) equipped with a blue laser set at 50 mW and an excitation wavelength of 488 nm. Multiparametric analyses were carried out on both scattering signals, including forward and side scatter, while SYBR Green I fluorescence was detected on the BL1 530/30 nm channel. Cell debris were excluded from the acquisition analysis by setting a BL1 threshold. In addition, the gated fluorescence events were evaluated on the forward-sideways density plot to exclude background events and to obtain an accurate microbial cell count, as previously described (Vandeputte et al., 2017).

### 2.5 Growth assays using co-culture duet system

To investigate the interactions among co-culture members and validate the proof of concept, we employed a Cerillo co-culture duet system (Cerillo, USA). In this system, the wells are separated by a semi-permeable membrane divider filter with a porosity of 0.2 μm, allowing for the monitoring individual strain cell numbers by flow cytometry within the co-culture. The experiment was performed in triplicate.

### 2.6 RNA extraction and sequencing

Total RNA was isolated as previously described (Turroni et al., 2010). Briefly,1 mL of QIAZOL (Qiagen, United Kingdom) and 0.8 g of glass beads (diameter, 106 μm; Sigma) were used to resuspend cell pellets for lysis. Specifically, this was obtained by alternating 2 min of stirring on a bead beater with 2 min of static cooling on ice. RNA was recovered from the upper phase after centrifugation at 12,000 rpm for 15 min and after purification using an RNeasy minikit (Qiagen, Germany) following the manufacturer's guidelines. The RNA concentration and purity were evaluated using a spectrophotometer (Eppendorf, Germany). For RNA-Seq, from 100 ng to 1 μg of extracted RNA was depleted from rRNA by employing QIAseq FastSelect−5S/16S/23S according to the producer's guide (Qiagen, Germany). mRNA yield was checked using a Tape station 2200 (Agilent Technologies, USA). Then, the TruSeq Standard mRNA preparation kit (Illumina, San Diego, CA) was used to prepare the sequencing library. The samples were run into a NextSeq 2000 high output v2.5 kit (150 cycles, single end; Illumina). Low-quality reads (minimum mean quality, 20; minimum length, 150 bp) and any residual ribosomal locus-encompassing reads were removed using the METAnnotatorX2 (Milani et al., 2021). Bowtie2 software (Langdon, 2015) was used to align the reads with respect to the reference genome of each *Bacteroides* strain used. Htseq-counts script of HTSeq software in “union” mode was employed to quantify reads mapped to individual transcripts (Anders et al., 2015). Raw data were then normalized utilizing CPM (mapped reads) for filtering genes with low counts (CPM < 1) and trimmed mean of *M* values (TMM) for statistically robust differential gene expression analysis through the EdgeR package (Robinson et al., 2010). Genes showing a fold-change in transcription of ≥2 (Log_2_FC > 1; up-regulated) and a fold-change in transcription of ≤ 2 (Log_2_FC < 1; down-regulated), in combination with a *p*-value of ≤ 0.05 calculated through correction for multiple comparisons using the False Discovery Rate (FDR) procedure, were considered as significantly differentially transcribed between the bi-associations when compared to mono-associations.

### 2.7 Statistical analyses

All statistical analyses were performed using SPSS software, except for beta-diversity comparisons. Before group comparisons, the Shapiro-Wilk test was applied to assess the normality of the distribution for each variable, including bacterial species abundances, alpha-diversity indices, and enzyme-coding gene counts, within each sample. Based on the normality test results, parametric or non-parametric tests were selected accordingly. Specifically, ANOVA was used for normally distributed data, while the Kruskal-Wallis test was applied for non-normally distributed data. *Post-hoc* pairwise comparisons between treatment groups were performed using the Bonferroni correction to control for multiple testing. These tests evaluated alpha-diversity differences, bacterial taxa's relative abundances, and functional genes across experimental conditions. Differences in beta-diversity between groups were assessed using PERMANOVA (Permutational Multivariate Analysis of Variance), based on Bray–Curtis dissimilarity matrices, computed with QIIME2 (Bolyen et al., 2019) using 999 permutations. Moreover, MaAsLin2 software (Mallick et al., 2021) was used to perform multivariable association analysis to evaluate the impact of botanical treatments (AVG and DR) on microbial taxonomic profiles while accounting for inter-individual variability. In detail, a linear mixed-effects model was applied, specifying treatment (AVG, DR) as a fixed effect and sample identity (AGM1–AGM10) as a random effect. The CTRL group was set as the reference condition. The Benjamini–Hochberg false discovery rate (FDR) correction adjusted *P*-values for multiple testing.

## 3 Results

### 3.1 Assessing the influence of aloe vera gel and dandelion root extract on gut microbiota composition

**Figure 1:**
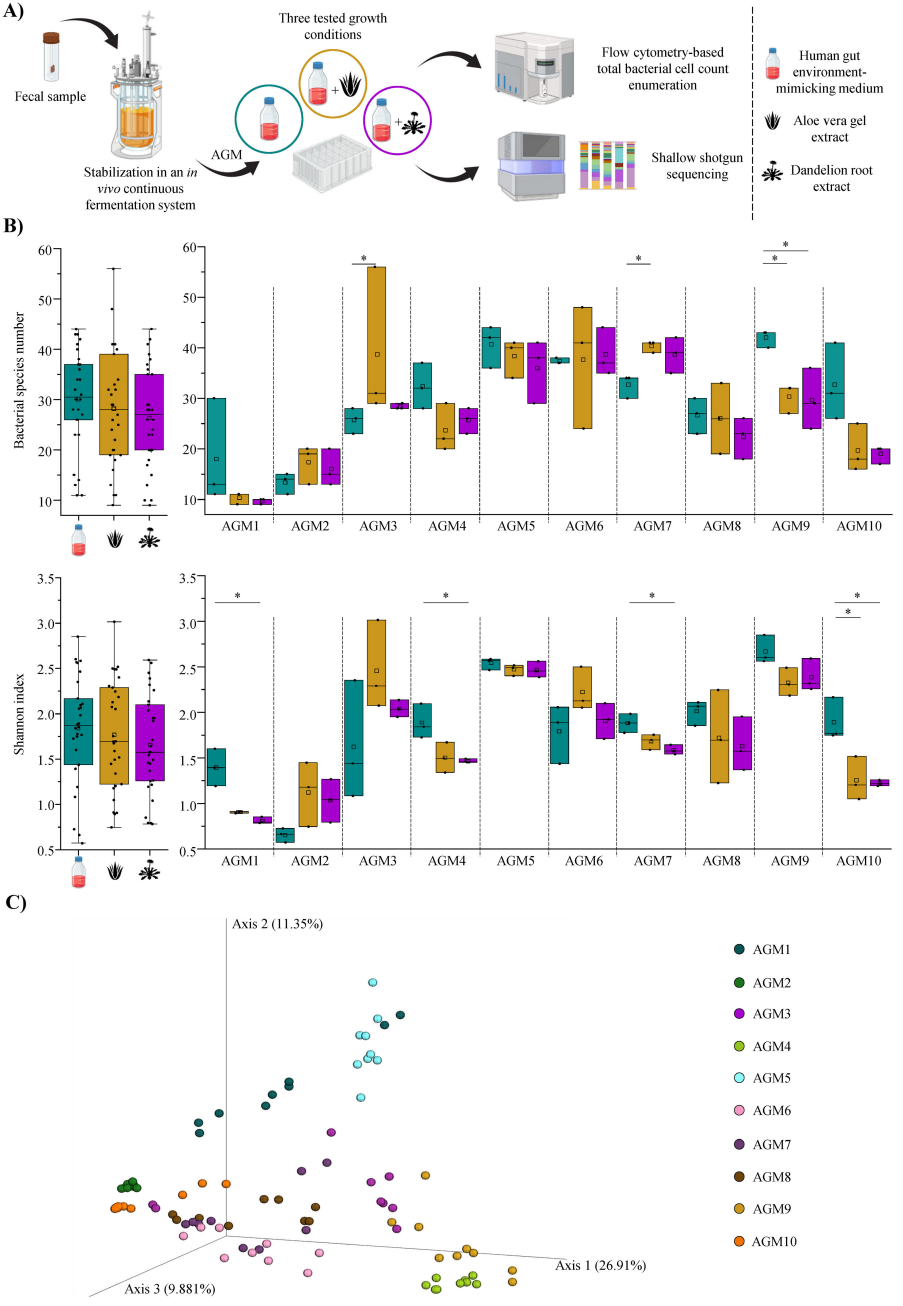
Plant extracts-driven modulation of the human gut microbiota biodiversity. **(A)** Schematically depicts the workflow used to assess the modulatory effects of AVG and DR extracts on the human gut microbiota. **(B)** Shows the box and whiskers plots of the alpha-diversity evaluated through a species richness analysis (at the top) and the Shannon index calculation (at the bottom). In both cases, the graphs on the left report the overall species richness or Shannon index average for each group, i.e., control samples and AVG- and DR-treated samples, while the graphs on the right display the two alpha-diversity indices calculated as the average of the obtained triplicates for each AGM and condition. In all cases, boxes are determined by the 25th and 75th percentiles, while the maximum and minimum values determine the whiskers and correspond to the box's extreme values. Lines inside the boxes represent the average, while squares correspond to the median. **(C)** Depicts the three-dimensional Bray-Curtis dissimilarity index-based PCoA of each sample divided per AGMs. *p < 0.05.

To investigate whether AVG or DR extracts may exploit a role in modulating the human intestinal ecosystem, 10 AGMs were cultured in the presence of each botanical extract as well as in their absence as control samples in a 96-deep well plate, as previously described (Li et al., 2019; Alessandri et al., 2022; Figure 1A). The AGMs were obtained in the framework of another study through a continuous cultivation system, starting from fecal samples of healthy adult human individuals as inoculum, allowing them to stabilize both dominant and accessory bacterial species of 10 different subjects (Alessandri et al., 2024). Specifically, except for AGM1 and AGM2, which resulted in being dominated by *Escherichia coli*, for the other eight AGMs, a predominance of the genus *Bacteroides*, i.e., one of the most abundant and prevalent bacterial taxa of the human gut microbiota (Arumugam et al., 2011; Mancabelli et al., 2017; Costea et al., 2018; Alessandri et al., 2024; Shin et al., 2024). In addition, other main microbial players of the human gut microbiota, including *Akkermansia muciniphila, Anaerotignum faecicola, Collinsella aerofaciens, Faecalibacterium prausnitzii, Parabacteroides distasonis, Parabacteroides merdae, Phocaeicola dorei*, and *Ruminococcus gnavus*, recently reclassified as *Mediterraneibacter gnavus* (Togo et al., 2018) were also stabilized.

Cultivations of AGMs in presence of botanicals were carried out in triplicate by exploiting the identical human gut environment-simulating culture medium used for the stabilization of the AGMs (Alessandri et al., 2022, 2024). After 24 h of growth under anaerobic conditions, AGMs cultures were subjected to DNA extraction and shallow shotgun sequencing (Figure 1A). The latter produced 5,661,100 quality-filtered reads with an average of 62,901 filtered reads per sample (Supplementary Table S1).

The generated species-level taxonomic profiles were used to explore the α-diversity among samples. Specifically, an overall species richness analysis did not reveal any significant difference in the number of detected bacterial species among the three groups, i.e., control and AVG- or DR-treated samples (ANOVA Bonferroni *p*-value > 0.05 in all cases), as also confirmed by the calculation of the Shannon index (Figure 1B). Thus, this suggests that these botanicals do not induce drastic/major shifts in the bacterial complexity of the gut microbiota. A desirable outcome since a radical alteration of gut bacterial species number is generally associated with dysbiosis status (Scher et al., 2015; Chandrasekaran et al., 2023; Hajj Hussein et al., 2023; Jauregui-Amezaga and Smet, 2024). However, the same species richness analysis performed for each AGM highlighted some significant variations (Figure 1B). Indeed, both AGM3 and AGM7 showed a meaningful enhancement of the bacterial complexity when AGMs were exposed to AVG with respect to the control (ANOVA Bonferroni *p*-value of 0.036 and 0.029, respectively; Figure 1B). For AGM9, a significant decrease in bacterial taxa was recorded in the presence of both AVG and DR when compared to the control (ANOVA Bonferroni *p*-value of 0.035 and 0.027, respectively; Figure 1B). Similarly, the Shannon index calculation highlighted an important reduction of the α-diversity for AGM1, AGM4, and AGM7 when exposed to DR (ANOVA Bonferroni *p*-value < 0.05 in all cases), as well as in AGM10 when cultivated in the presence of both AVG and DR when compared to the controls (ANOVA Bonferroni *p*-value of 0.021 and 0.017, respectively; Figure 1B). This suggests that, in certain circumstances (in 35% of cases), AVG and DR may exert a role in modulating the bacterial complexity of the human gut microbiota through specific microbial taxa.

Interestingly, no significant compositional differences were observed among the three different groups within each sample (pairwise PERMANOVA *p*-value > 0.05 in all cases) using a β-diversity analysis based on a Bray-Curtis dissimilarity matrix (Figure 1C). Indeed, no treatment-associated clustering of samples was observed. Thus, suggesting that neither AVG nor DR significantly modify the overall abundance ratios among bacterial species composing the human gut microbiota.

### 3.2 Aloe vera gel or dandelion root extract-driven modifications of the gut microbiota taxonomic composition

**Figure 2:**
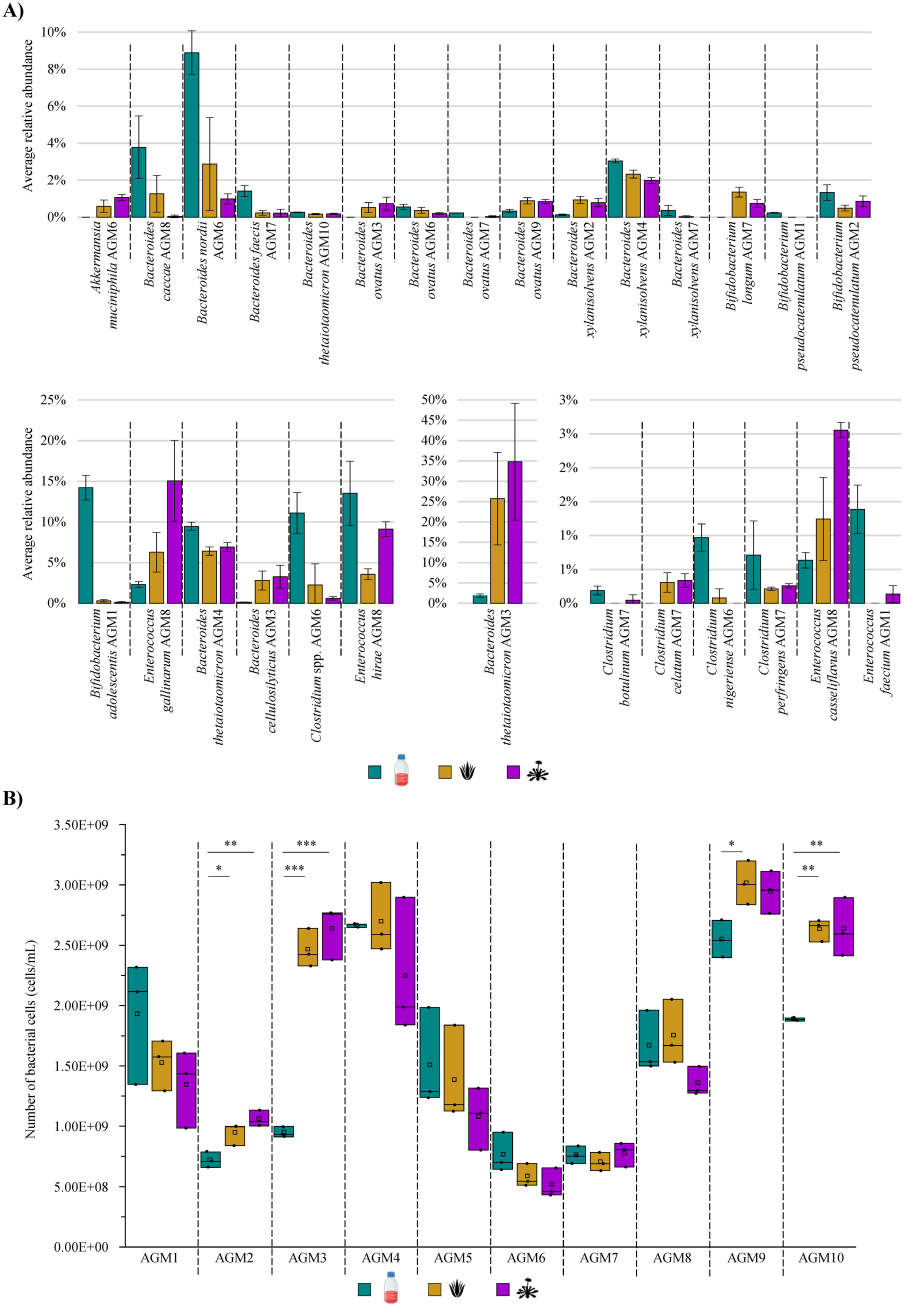
Modulation of gut microbiota taxonomic composition and growth performances in the presence of AVG and DR. **(A)** Reports the average relative abundance of the bacterial species that significantly differ among the three tested growth conditions per AGM. **(B)** Displays the box and whiskers plot associated with the total bacterial cell enumeration of each AGM in tested growth conditions. In the plot, boxes are determined by the 25th and 75th percentiles, while the whiskers are determined by the maximum and minimum values and correspond to the box extreme values. The lines inside the boxes represent the average, while the squares correspond to the median. *p < 0.05; **p < 0.01; ***p < 0.001.

To evaluate whether AVG or DR extracts may have a role in modulating the abundance of the various bacterial taxa constituting the human gut microbiota, any significant differences were investigated for each sample among the three groups. In this context, a total of 40 bacterial species showed key changes in their relative abundance, suggesting that these botanicals may have an effective role in influencing the abundance ratios among the bacterial taxa of the intestinal microbiota (Figure 2A and Supplementary Table S2). In-depth insights into the bacterial species whose abundance significantly differed among the conditions tested revealed that the relative load of *Akkermansia muciniphila* underwent a significant increase in AGM6 when in the presence of both AVG and DR with respect to the reference conditions (ANOVA Bonferroni *p*-value of 0.047 and 0.003, respectively; Figure 2A and Supplementary Table S2). This microorganism has recently been proposed as a novel promising probiotic bacterium due to its ability to attenuate certain inflammatory conditions, including acute and chronic colitis, or improve metabolic disorders, while its absence has been demonstrated to be associated with a plethora of diseases such as diabetes, obesity, or cancer (Bian et al., 2019; van der Lugt et al., 2019; Segers and de Vos, 2023; Calvo et al., 2024; Jiang et al., 2024; Panzetta and Valdivia, 2024). Therefore, since AVG and DR stimulate the abundance of *A. muciniphila* in AGM6, it can be argued that these botanicals may exert a potential beneficial effect on the intestinal microbiota, contributing to improving the abundance of this positive bacterium and, therefore, the overall host health. However, in the other sample containing this bacterial species, i.e., AGM5, *A. muciniphila* underwent no significant change (Supplementary Table S2). Therefore, this suggests that the ability of these botanicals to promote an increase in the relative abundance of these beneficial bacterial species may depend on cross-feeding interactions that originate in every AGM. It is plausible that some strains may possess genetic traits that encode enzymes that degrade these two plant extracts, allowing for energy recovery and the release of simpler compounds usable by other species (e.g., *A. muciniphila*) with which they share the ecological environment (Belzer et al., 2017; Wu et al., 2024).

In addition, members of another bacterial taxon known to exploit multiple beneficial effects upon its host, i.e., the genus *Bifidobacterium* (Bottacini et al., 2017; Hidalgo-Cantabrana et al., 2017; Alessandri et al., 2019; Martin et al., 2023), underwent significant shifts in their relative abundance when exposed to the two botanical extracts. Specifically, *Bifidobacterium adolescentis* exposed to DR in AGM1 (Kruskall-Wallis *p*-value of 0.034) and *Bifidobacterium pseudocatenulatum* cultivated in AVG for AGM2 (ANOVA Bonferroni *p*-value of 0.049) displayed a significant reduction in their relative abundances when compared to the control (Figure 2A and Supplementary Table S2). Conversely, *Bifidobacterium longum* loads significantly increased in AGM7 in the presence of both botanicals compared to the reference condition (ANOVA Bonferroni *p*-value < 0.05 in both cases; Figure 2A and Supplementary Table S2). Therefore, while for some bifidobacterial species, the two botanicals seemed to have a bacteriostatic/growth inhibitory effect, for other species, they may act as a selective substrate for their growth, as previously observed for other plant extracts (Duda-Chodak, 2012; Firrman et al., 2016; Santhiravel et al., 2022). Thus, it suggests that both AVG and DR can be exploited for the selective growth of particular bifidobacterial species with positive effects on the host health, given the renowned bifidobacterial anti-inflammatory properties (Martorell et al., 2021; Choi et al., 2022; Lueschow et al., 2022; Gavzy et al., 2023; Leser and Baker, 2023; Martin et al., 2023).

Beyond these potentially beneficial bacteria that generally represent only a small portion of the more complex and intricate human gut microbiota (Lay et al., 2005; Derrien et al., 2008; O'Callaghan and van Sinderen, 2016; Si et al., 2022), the analysis of the species-level taxonomic profiles revealed that AVG and DR also significantly influenced the relative abundance of species belonging to one of the most abundant and prevalent bacterial genera of the human gut microbiota, i.e., the genus *Bacteroides* (Arumugam et al., 2011; Mancabelli et al., 2017; Costea et al., 2018; Mancabelli et al., 2024). Specifically, *Bacteroides caccae* in AGM8 and *Bacteroides nordii* in AGM6, both treated with DR (ANOVA Bonferroni *p*-value of 0.02 and Kruskall-Wallis *p*-value of 0.02, respectively), showed a significant decrement in their relative abundance compared to the control. A similar reduction was observed for *Bacteroides faecis* in AGM7, also treated with both DR and AVG (ANOVA Bonferroni *p*-value < 0.05 in both cases). Conversely, *Bacteroides cellulosilyticus* in AGM3, treated with DR, displayed an opposite trend (ANOVA Bonferroni *p*-value of 0.03; Figure 2A and Supplementary Table S2). For three other *Bacteroides* species, instead, a significant change in their relative abundance with alterations varying according to the AGM they belong to was recorded (Figure 2A and Supplementary Table S2). Specifically, *Bacteroides ovatus* levels significantly increased in AGM3 with DR (ANOVA Bonferroni *p*-value of 0.04) and AGM9 with both AVG and DR (ANOVA Bonferroni *p*-value < 0.05 in both cases). At the same time, the species showed a significant reduction in AGM6 with DR (ANOVA Bonferroni *p*-value of 0.04) and AGM7 with AVG (Kruskall-Wallis *p*-value of 0.04) when compared to the reference condition (Figure 2A and Supplementary Table S2). Similarly, AGM3-associated *Bacteroides thetaiotaomicron* increased its relative abundance when cultured in DR (ANOVA Bonferroni *p*-value of 0.03), while the levels of this species significantly decreased in AGM4 and AGM10 in the presence of both AVG and DR when compared to the control (ANOVA Bonferroni *p*-value < 0.05 in all cases; Figure 2A and Supplementary Table S2). Finally, *Bacteroides xylanisolvens* relative abundance significantly increased in AGM2 after DR and AVG exposure (ANOVA Bonferroni *p*-value < 0.05 in both cases). At the same time, it decreased in AGM4 (ANOVA Bonferroni *p*-value < 0.05 in both cases) and AGM7 (Kruskall-Wallis *p*-value of 0.04 when compared to CTRL and DR; Figure 2A and Supplementary Table S2). Thus, suggesting not only that these two botanical extracts have a role in modulating the abundance of some of the most abundant and prevalent species of the human gut microbiota but also reinforcing the notion that these modifications in the abundance may be either strain-dependent or dependent on cross-feeding events closely associated with the gut microbiota composition.

Similar to *Bacteroides*, a different trend was observed for certain members of the genus *Enterococcus*, depending on the species. Indeed, *Enterococcus casseliflavus*, as well as *Enterococcus gallinarum*, and *Enterococcus* spp. significantly increased their load in AGM8 (Kruskall-Wallis *p*-value of 0.02, ANOVA Bonferroni *p*-value of 0.008 and 0.0008, respectively), while *Enterococcus faecium* and *Enterococcus hirae* average relative abundance decreased in AGM1 (Kruskall-Wallis *p*-value of 0.03) and AGM8 (ANOVA Bonferroni *p*-value of 0.007), respectively, when cultivated in the presence of one of the two botanicals with respect to the control (Figure 2A and Supplementary Table S2). On the other side, several species of the genus *Clostridium*, except for *Clostridium celatum*, showed a significant decrease in their relative abundance in the presence of at least one of the two botanicals (Figure 2A and Supplementary Table S2). In this context, since members of the genus *Clostridium* have been described as potential pathogens positively associated with gastrointestinal cancers, the AVG- and/or DR-driven decrease of these species can be considered a positive effect induced by these two botanicals (Yu et al., 2023; Cao et al., 2024; Garvey, 2024). These results show not only that the two botanicals are able to modulate the abundance ratios among species of the human intestinal microbiota, but they also appear to have a beneficial impact on the host health by promoting the growth of particular species with health-promoting activity and, at the same time, limiting/reducing the proliferation of other potentially pathogenic bacterial taxa.

To better understand the overall impact of botanical treatments on gut microbiota composition, a global statistical approach was implemented to evaluate treatment-driven effects while controlling for individual variability. Specifically, a linear mixed-effects model was applied using MaAsLin2, where treatments, i.e., AVG and DR, were included as fixed effects and sample identity was set as a random effect. This approach allowed the estimation of treatment effects across all samples, using the CTRL group as the reference condition.

At a significance threshold of *p* < 0.05, a total of 53 taxa showed treatment-associated changes in relative abundance (Supplementary Table S3). However, after correction for multiple tests using the Benjamini–Hochberg procedure (*q* < 0.05), only seven taxa remained significant (Supplementary Table S3). Each microbial taxon was counted only once, even when significantly affected by both treatments. Specifically, six bacterial taxa were significantly associated with AVG exposure and five with DR, all displaying negative coefficients. This observation suggests that both botanical extracts may selectively reduce the abundance of specific microbial taxa without inducing broad-scale shifts in overall community diversity.

This pattern aligns with the absence of significant changes in alpha-diversity, supporting the hypothesis that these botanicals exert targeted effects on particular taxa rather than altering the overall richness or evenness of the gut microbiota.

### 3.3 Impact of AVG and DR on gut microbiota growth performances

In addition to the fact that AVG- and/or DR-induced significant changes in the relative abundance of bacterial species composing the human gut microbiota, it was also assessed whether the two considered botanical extracts could have a role in modulating the growth performances of this complex microbial ecosystem. In this context, to obtain a comprehensive biological interpretation of AVG and DR effects on gut microbiota, a quantitative microbiome profiling assay was performed through flow cytometry to enumerate bacterial cells of each biological replicate and culture condition after 24 h of cultivation in a 96-deep well plate. Notably, considering the three macro-groups, no significant differences in the number of bacterial cells were observed among groups (Kruskal-Wallis *p*-value of 0.624), with a recorded average bacterial cell count of 1.54E+09 cells/mL, 1.77E+09 cells/mL, and 1.66E+09 cells/mL for the control, AVG- and DR-treated samples, respectively (Figure 2B). These data confirmed that the two botanicals produce no crucial effects either in stimulating or inhibiting the overall bacterial growth of the human intestinal microbiota. However, a thorough analysis of the statistical data obtained for each AGM revealed some significant differences in the growth performances of certain stabilized microbiota in the presence of AVG and/or DR compared to the control (Figure 2B). In detail, the number of bacterial cells was significantly higher after 24 h of cultivation in AGM2, AGM3, and AGM10 in the presence of either AVG or DR when compared to the control (ANOVA Bonferroni *p*-value < 0.05 for all cases; Figure 2B). In addition, the exposure to AVG extract induced a significant bacterial cell number increment in AGM9 with respect to the control (ANOVA Bonferroni *p*-value = 0.047; Figure 2B). This notion suggests that, depending on the taxonomic composition of the intestinal microbiota, the two plant extracts may have a role in influencing the growth performances of (some) bacterial species composing the human gut microbiota.

### 3.4 Cross-feeding activity on *Bacteroides* species

**Figure 3:**
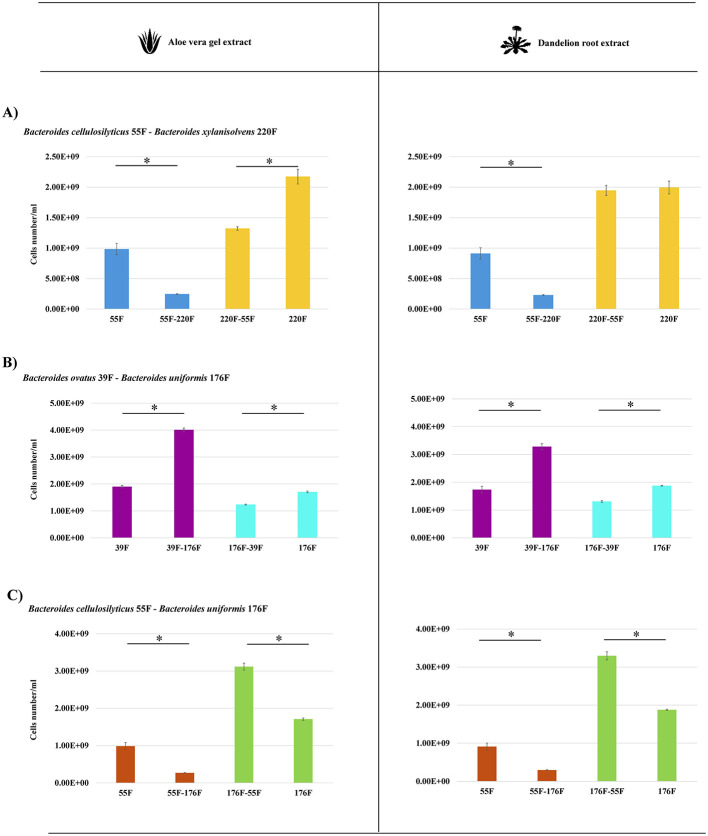
Quantification of the cell numbers of *Bacteroides* species in bi-association experiments. **(A)** depicts the cell number evaluation of the *Bacteroides cellulosilyticus* 55F and *Bacteroides xylanisolvens* 220F growth in mono and bi-association on AVG on the left and DR on the right. **(B)** Displays the cell number evaluation of the *Bacteroides ovatus* 39F-*Bacteroides uniformis* 176F growth in mono and bi-association on AVG on the left and DR extract on the right. In **(C)** the cell number quantification of the *Bacteroides cellulosilyticus* 55F and *Bacteroides uniformis* 176F growth in mono and bi-association on AVG on the left and DR extract on the right were shown. The flow cytometry quantification results are represented by bars in which the *y*-axis is the number/ml of bacterial cells, and the *x*-axis shows the names of the strains involved in mono-and bi-associations. Asterisks indicate that the presented data display a significant (*p < 0.0003) deviation from the obtained values of the mono-association.

**Figure 4:**
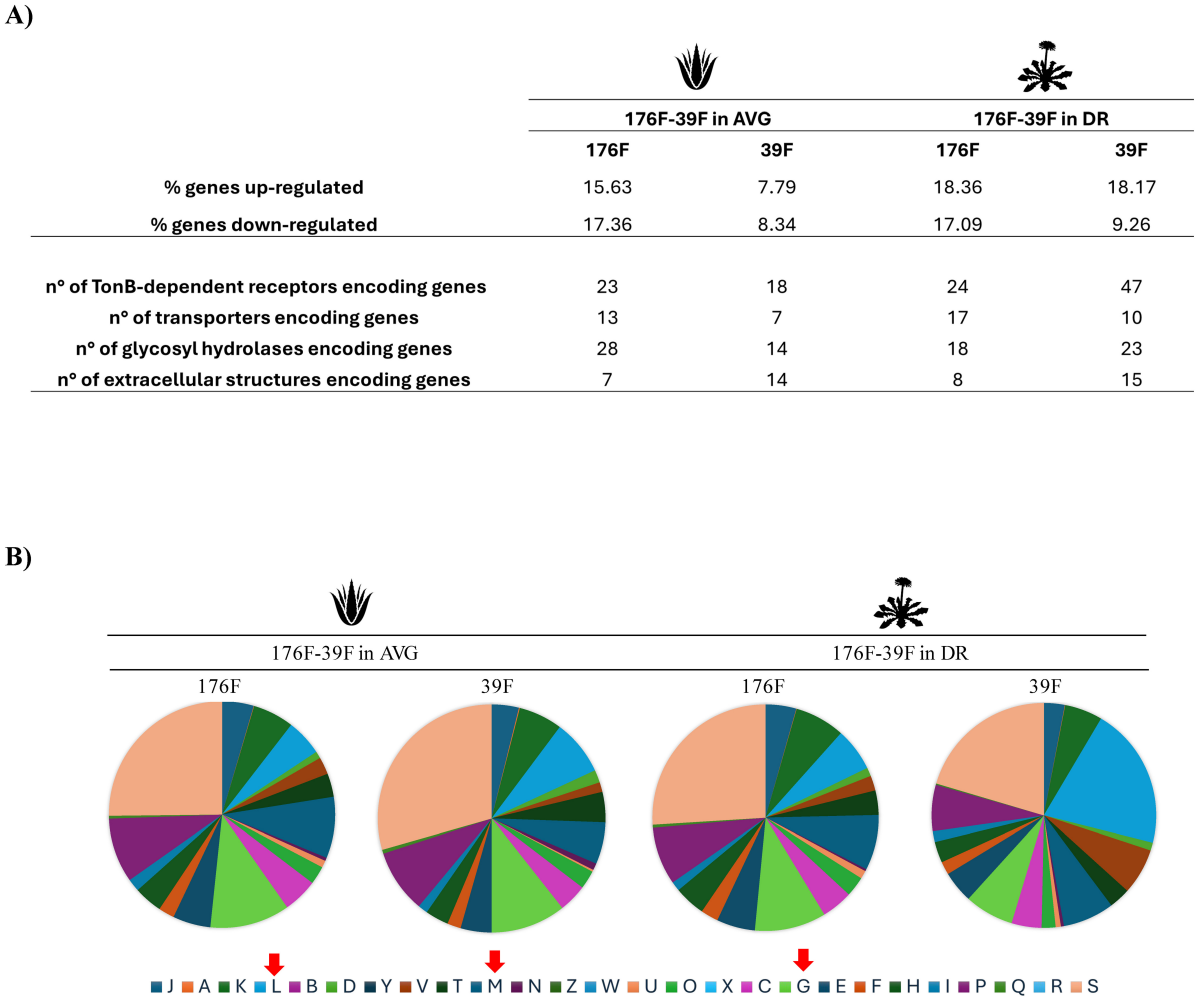
Effects of AVG and DR on *Bacteroides* transcriptomes after cross-feeding experiments. **(A)** displays the percentage of genes up and down-regulated of the two strains *Bacteroides ovatus* 39F and *Bacteroides uniformis* 176F, during their bi-association in AVG on the left and DR on the right. **(B)** depicts cakes diagram of the statistically significant impacted genes (up- and down-regulated) of the two strains' growth in bi-association subdivided following the Cluster of Orthologue Genes (COG) classification. The red arrows indicate the most represented classes.

The *Bacteroides* species, representing the dominant taxa of 8 of the 10 AGMs, appears to be significantly impacted by botanicals, i.e., seven different species significantly increase/decrease their cell numbers (Figure 2A). To corroborate whether bi-associations of *Bacteroides* species could engage cross-feeding interactions on AVG and DR extract, respectively, *in*-*vitro* duets co-culture assays were established in triplicates. Specifically, the number of bacterial cells of the couple *Bacteroides cellulosilyticus* 55F-*Bacteroides xylanisolvens* 220F, *Bacteroides cellulosilyticus* 55F-*Bacteroides uniformis* 176F and *Bacteroides ovatus* 39F-*Bacteroides xylanisolvens* 220F were enumerated by flow cytometry. Growth analyses showed that the presence of botanicals has an impact in all the bi-associations except only for *B. xylanisolvens* 220F on DR, which appears to be unaltered (Figure 3A). On the contrary, *B. ovatus* 39F cells showed a significant enhancement when co-cultivated with *B. uniformis* 176F (*p*-value < 0.0001) both in the presence of AVG and DR, respectively (Figure 3B). The same behavior was depicted for *B. uniformis* 176F when grown in the presence of *B. cellulosilyticus* 55F (*p*-value equals 0.0001) in the presence of AVG and DR, respectively (Figure 3C). This data reinforces the assumption highlighted in previous experiments that the gut microbiota responds to botanicals differently, strongly dependent on the original composition of the complex microbial communities. Furthermore, the impact on gene expression of these *Bacteroides* strains grown in bi-association, which were highly affected by the presence of botanicals compared to single cultivation, was evaluated through RNA sequencing. The latter generated 238474 quality-filtered reads, averaging 9936 reads per sample (Supplementary Table S4). In this context, only genes showing a fold-change in the transcription of ≥2 (Log_2_FC > 1) in combination with a *p*-value of ≤ 0.05 calculated through correction for multiple comparisons using the False Discovery Rate (FDR) procedure were considered as significantly differentially transcribed between the bi-associations when compared to mono-associations. Interestingly, strains grown in bi-association were found to have an increase in gene expression ranging from 7.79 to 18.36% compared to growth alone, suggesting different cross-feeding interactions (Figure 4A). According to the Cluster of Orthologous Genes (COGs) categorization, the most impacted genes are involved in replication/recombination/repair (L), cell wall/membrane/envelope biogenesis (M), and carbohydrate transport and metabolism (G) in addition to the function unknown (S) (Figure 4B). These data clearly show that the strains are metabolically more active in bi-association and that the external structures involved in the uptake and metabolism of carbon sources are highly expressed in such conditions. There are several genes encoding glycoside hydrolases, glycan-binding surface proteins, sugar transferases, ABC and MFS transporters, cell surface proteins, and secretion systems that are up-regulated when bacteria are co-cultivated on these botanicals respect when they are grown alone (Figure 4A and Supplementary Tables S5–S8). In addition, several TonB-dependent receptors are expressed when the strains are growing in bi-association, with respect to the mono-association, ranging from 18 to 47 genes. TonB-dependent receptors comprise outer membrane transport systems that could be involved in the transport of several substrates, such as proteins, inorganic substances (e.g., iron), vitamins, starch, and lignin-derived aromatic compounds (Noinaj et al., 2010; Fujita et al., 2019). All these data reinforce the notion that during bi-association, a cross-talk occurs between the involved strains in the presence of botanicals.

### 3.5 Prediction of AVG- and/or DR-driven functional changes in the human gut microbiome

Shotgun sequencing data allowed us to assess whether taxonomic changes were also conveyed by significant alterations in the functional potential of the human gut microbiota in terms of enzymatic profiles based on the MetaCyc database and Enzymatic Commission classification. Interestingly, a variable number of enzyme-encoding genes whose abundance significantly differed among the three tested groups per AGM was observed, ranging from 6 in AGM5 to 47 in AGM10 (Supplementary Table S9). Thus, this confirms that the taxonomic botanical-induced changes are also associated with metabolic modifications and suggests that the latter are, as expected, gut microbiota taxonomic composition-dependent.

Furthermore, in-depth insights into the microbiome data, whose abundance varied significantly between control samples and those exposed to botanical extracts, revealed that, in most cases, they corresponded to genes encoding enzymes involved in vitamin and carbohydrate metabolism, as well as even if to a less extent, in pathways associated with the production of SCFA, antibiotics, amino acid or butanoate metabolism (Supplementary Table S9 and Supplementary text). Thus, it leads to the notion that plant extracts influence the taxonomic composition of the gut microbiota by mostly favoring changes in the abundance of genes involved in these specific metabolic pathways. However, a unique modification pattern in the abundance of genes participating in the same metabolic pathway was not observed among AGMs, corroborating the data about AVG and DR's effects on the gut microbiota composition.

## 4 Discussion

The human gut harbors a diverse microbial community that supports overall health by aiding digestion, generating essential metabolites, and regulating the immune system. However, disruptions to this microbial balance can contribute to various health problems, including gastrointestinal diseases and metabolic disorders. Functional vegetables rich in bioactive compounds such as polyphenols and flavonoids have positively affected gut health and may help mitigate dysbiosis. Among these, AVG and DR stand out due to their traditional medicinal use for their antioxidant and anti-inflammatory properties. Nonetheless, their specific effects on the gut microbiota's taxonomic composition and functional capabilities remain insufficiently understood.

In this study, the growth of 10 artificial gut microbiota (AGM) communities cultivated in a bioreactor, exposed to the botanical extracts and not as controls, showed no drastic changes among the three groups, i.e., control and AVG- or DR-treated samples, if we consider the 10 AGMs as biological replicates. However, a more detailed analysis of each sample revealed significant changes in the relative abundance of 40 bacterial species, suggesting that these botanicals may substantially impact modulating the composition of the intestinal microbiota. Some of these microbial genera are associated with good gut balance, i.e., *Bifidobacterium, Akkermansia*, and *Bacteroides*, highlighting a possible positive impact on consumer health. The effect could be due to the growth/reduction of some microorganisms that, in turn, influence others, like the network in a mesh, highlighting how each microorganism in a complex microbiota has an impact on all the others, which appears to be dependent on the taxonomic composition of the intestinal microbiota exposed to the two botanicals. This behavior is confirmed by specific cross-feeding experiments carried out on some species of the *Bacteroides* genus, which resulted statistically impacted in their growth. This observation reinforces that such botanicals exploit a molecular effect on the human gut microbiota members, indirectly influencing human health. This is in line with what the World Health Organization already emphasizes in preferring a healthy diet, that is to favor plant-based foods rather than simple sugars, salt, and fats (https://www.who.int/news-room/fact-sheets/detail/healthy-diet). Indeed, further investigation into how the presence of botanicals impacts the specific microbial taxa observed in this study would help to understand and explain the dynamics observed in our complex microbiota experiments.

## Data Availability

Raw shallow shotgun sequences and RNAseq data are accessible through the Sequence Read Archive (SRA) under the BioProject accession number PRJNA1247491.
