# Supplementary material for: Dissecting the molecular interactions between botanical extracts and the human gut microbiota
Source: Front Microbiol. 2025 Jul 16;16:1610170. doi: 10.3389/fmicb.2025.1610170 (PMC12307401; doi:10.3389/fmicb.2025.1610170)
Supplement: Supplementary file 1 [file Table_1.docx]

**Supplementary text**

**Detailed AVG- and/or DR-driven functional changes in the human gut microbiota.**

While genes coding for enzymes involved in thiamine (B1 vitamin) metabolism pathway were significantly enriched in AGM1, AGM3, AGM8, and AGM10 as well as in AGM2 and AGM7 control samples when compared to the corresponding AVG and DR treated ones, respectively, the abundance of other genes participating to the same metabolic pathway resulted to be significantly reduced in AGM2, AGM3, and AGM4 control samples with respect to those exposed to AVG (Table S9). Similarly, the abundance of the predicted genes coding for an enzyme involved in niacin (B3 vitamin) metabolism was significantly higher in the AGM8 controls when compared to the AVG-treated samples, while an opposite trend was observed for another gene belonging to the same pathway in AGM9 (Table S9). At the same time, in AGM1 and AGM8, different genes involved in folate (B9 vitamin) metabolism were enriched in the presence of AVG or DR when compared to the control, while a significantly reduced abundance was observed for other genes in AGM4 and AGM6 (Table S9). Conversely, the abundance of genes involved in riboflavin (B2 vitamin) and pantothenate (B5 vitamin) metabolism were significantly enriched only in the control samples of various AGMs when compared to their counterpart exposed to AVG or DR (Table S9).

Furthermore, fluctuating differences were also observed for genes involved in carbohydrate metabolism (Table S9). However, while control samples of different AGMs were characterized by a significant increase in the abundance of genes involved in the degradation or modification of simple sugars, except for a cellulase in the AGM2 control samples when compared to the DR group, in the case of samples exposed to botanicals, instead, an increase in the abundance of some genes involved in complex carbohydrate breakdown was recorded (Table S9). Probably, since a significant fraction of the botanical bioactive compounds is represented by complex (botanical-specific) polysaccharides (Xie et al., 2016; Cai et al., 2019; Liu et al., 2019; Ignat et al., 2021; Khan et al., 2022; Li et al., 2022), the presence of the latter in the culture medium may play a role in selecting bacterial species whose genome is enriched in genes responsible for their degradation.

Finally, a significantly higher abundance of genes involved in butanoate metabolism was recorded when AGM6 and AGM8 were exposed to AVG and DR, respectively, compared to the control (Table S9). Butanoate metabolism is strictly related to butyrate production, i.e., an SCFA with renowned health-promoting activities. Indeed, butyrate, the preferred energy source for intestinal epithelial cells, promotes tight junction reinforcement, intestinal cell proliferation, and mucin production by Goblet cells (Gaudier et al., 2004). In addition, this SCFA directly interacts with the host immune system towards an anti-inflammatory response (Mann et al., 2024). In this context, it can be suggested that the two selected plant extracts, having a role in implementing the abundance of genes associated with this positive SCFA, may have a role in enhancing the health of hosts.

**References**

Cai, L., Chen, B., Yi, F., and Zou, S. (2019). Optimization of extraction of polysaccharide from dandelion root by response surface methodology: Structural characterization and antioxidant activity. *Int J Biol Macromol* 140**,** 907-919. doi: 10.1016/j.ijbiomac.2019.08.161.

Gaudier, E., Jarry, A., Blottiere, H.M., de Coppet, P., Buisine, M.P., Aubert, J.P., et al. (2004). Butyrate specifically modulates MUC gene expression in intestinal epithelial goblet cells deprived of glucose. *Am J Physiol Gastrointest Liver Physiol* 287(6)**,** G1168-1174. doi: 10.1152/ajpgi.00219.2004.

Ignat, M.V., Coldea, T.E., Salanta, L.C., and Mudura, E. (2021). Plants of the Spontaneous Flora with Beneficial Action in the Management of Diabetes, Hepatic Disorders, and Cardiovascular Disease. *Plants (Basel)* 10(2). doi: 10.3390/plants10020216.

Khan, R.U., Naz, S., De Marzo, D., Dimuccio, M.M., Bozzo, G., Tufarelli, V., et al. (2022). Aloe vera: A Sustainable Green Alternative to Exclude Antibiotics in Modern Poultry Production. *Antibiotics (Basel)* 12(1). doi: 10.3390/antibiotics12010044.

Li, Y., Chen, Y., and Sun-Waterhouse, D. (2022). The potential of dandelion in the fight against gastrointestinal diseases: A review. *J Ethnopharmacol* 293**,** 115272. doi: 10.1016/j.jep.2022.115272.

Liu, C., Cui, Y., Pi, F., Cheng, Y., Guo, Y., and Qian, H. (2019). Extraction, Purification, Structural Characteristics, Biological Activities and Pharmacological Applications of Acemannan, a Polysaccharide from Aloe vera: A Review. *Molecules* 24(8). doi: 10.3390/molecules24081554.

Mann, E.R., Lam, Y.K., and Uhlig, H.H. (2024). Short-chain fatty acids: linking diet, the microbiome and immunity. *Nat Rev Immunol* 24(8)**,** 577-595. doi: 10.1038/s41577-024-01014-8.

Xie, J.H., Jin, M.L., Morris, G.A., Zha, X.Q., Chen, H.Q., Yi, Y., et al. (2016). Advances on Bioactive Polysaccharides from Medicinal Plants. *Crit Rev Food Sci Nutr* 56 Suppl 1**,** S60-84. doi: 10.1080/10408398.2015.1069255.
